# Supplementary material for: Variation in adult sex ratios in tetrapods is linked to sex chromosomes through mortality differences between males and females
Source: PLoS Biol. 2025 May 12;23(5):e3003156. doi: 10.1371/journal.pbio.3003156 (PMC12148232; doi:10.1371/journal.pbio.3003156)
Supplement: S1 Fig — On the axes, >0.5 sex ratio values mean male-skewed sex ratios and <0.5 values mean female-skewed sex ratios, while positive bias values mean higher male than female mortalities/maturation times, whereas negative bias values mean higher female than male mortalities/maturation times. Regression lines in the figures are calculated from PGLS models using non-standardized data. The data underlying this figure can be found in S1 Data, 10.6084/m9.figshare.28562399. Statistically significant relationships are illustrated with continuous lines, whereas not statistically significant relationships are shown with dashed regression lines (see S3 Table). For each panel, black regression lines are from models with all data points, and gray regression lines are from models without outlier data points (S2 Table). (PDF) [file pbio.3003156.s001.pdf]

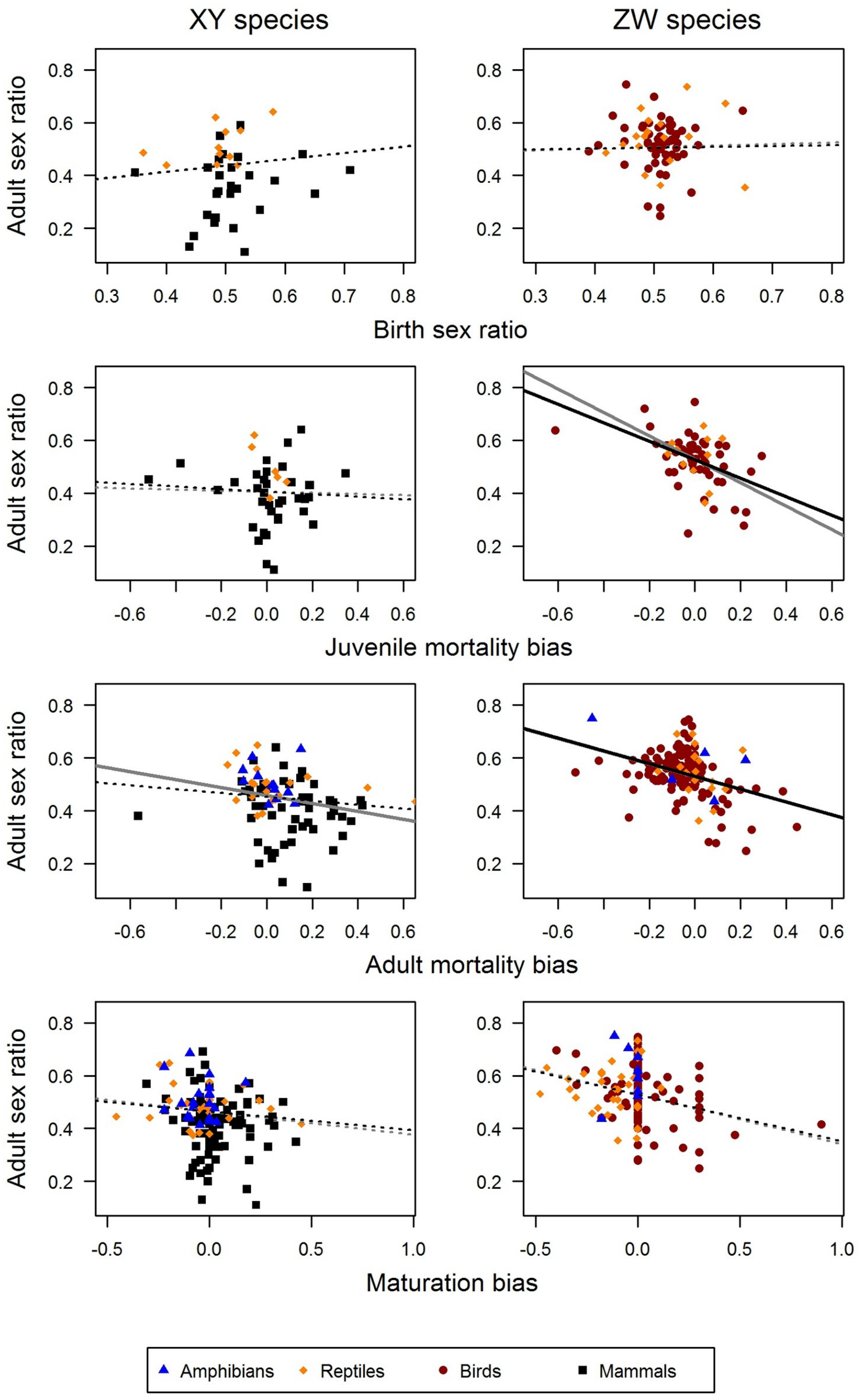

S1 Fig. Relationships of ASR with demographic traits (i.e. birth sex ratio, juvenile mortality bias, adult mortality bias, and maturation bias) in XY and ZW species separately. On the axes, >0.5 sex ratio values mean male-skewed sex ratios and <0.5 values mean female-skewed sex ratios, while positive bias values mean higher male than female mortalities/maturation times, whereas negative bias values mean higher female than male mortalities/maturation times. Regression lines in the figures are calculated from PGLS models using non-standardised data. The data underlying this figure can be found in S1 Data DOI: 10.6084/m9.figshare.28562399. Statistically significant relationships are illustrated with continuous lines, whereas not statistically significant relationships are shown with dashed regression lines (see S3 Table). For each panel, black regression lines are from models with all data points, and grey regression lines are from models without outlier data points (S2 Table).
