# Supplementary material for: Variation in adult sex ratios in tetrapods is linked to sex chromosomes through mortality differences between males and females
Source: PLoS Biol. 2025 May 12;23(5):e3003156. doi: 10.1371/journal.pbio.3003156 (PMC12148232; doi:10.1371/journal.pbio.3003156)
Supplement: S2 Fig — In (A), the tested combinations of potentially important direct (light gray arrows) and indirect (dark gray arrows) links from genetic sex determination to adult sex ratio are presented. Influence of the three demographic traits on adult sex ratio (black arrows) were included in all models. In (B), panels present the standardized path coefficients inferred by each supported model. Confidence intervals were estimated by bootstrapping, using the ‘boot’ function with 500 iterations. Statistics for the model fit and comparisons are presented in Table 2B in the main text. GSD, genetic sex determination; JMB and AMB, juvenile and adult mortality bias; MAT, maturation bias; ASR, adult sex ratio. The data and phylogeny underlying the analyses and results displayed in this figure can be found in S1 Data and S1 File, 10.6084/m9.figshare.28562399. (PDF) [file pbio.3003156.s002.pdf]

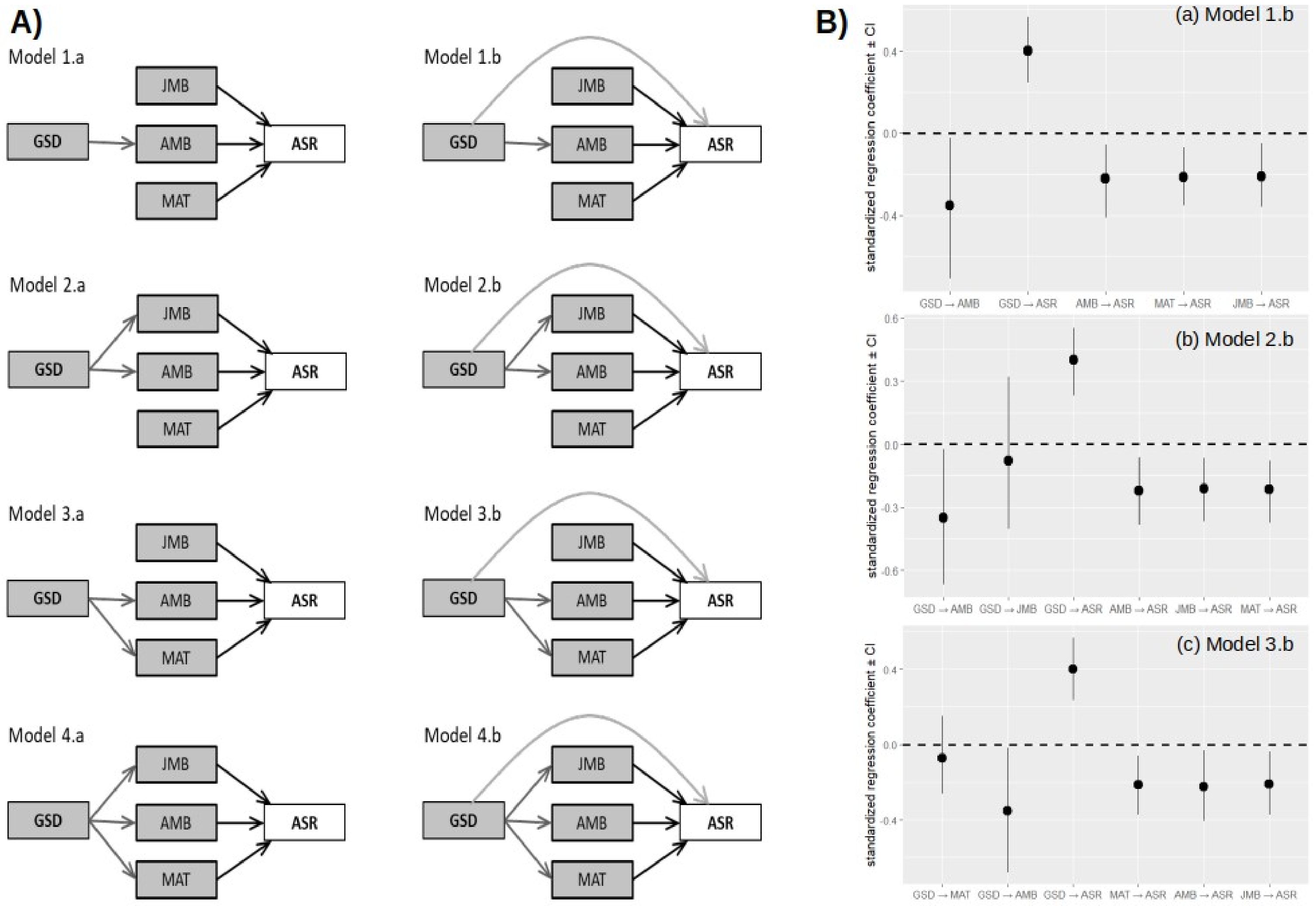

S2 Fig. **Visual representation of (A) our model set for path analysis, showing all models we tested, and (B) the standardised path coefficients and associated confidence intervals for the most supported models as inferred by analyses using R packge 'phylopath'.** In A), the tested combinations of potentially important direct (light grey arrows) and indirect (dark grey arrows) links from genetic sex determination to adult sex ratio are presented. Influence of the three demographic traits on adult sex ratio (black arrows) were included in all models. In B), panels present the standardized path coefficients inferred by each supported model. Confidence intervals were estimated by bootstrapping, using the 'boot' function with 500 iterations. Statistics for the model fit and comparisons are presented in Table 2B in the main text. GSD: genetic sex determination, JMB and AMB: juvenile and adult mortality bias, MAT: maturation bias, ASR: adult sex ratio. The data and phylogeny underlying the analyses and results displayed in this figure can be found in S1 Data and S1 File https://figshare.com/articles/dataset/S1_File_tetrapodtree_Nee_phy/28562399.
