# Supplementary material for: Variation in adult sex ratios in tetrapods is linked to sex chromosomes through mortality differences between males and females
Source: PLoS Biol. 2025 May 12;23(5):e3003156. doi: 10.1371/journal.pbio.3003156 (PMC12148232; doi:10.1371/journal.pbio.3003156)
Supplement: S3 Fig — We used the ‘make.simmap’ function in the ‘phytools’ package in R with 100 simulations. Result of the “all rates different” (ARD) model type is shown, where different transition rates are allowed between the states, because this model type was supported most by its AIC value. Pie charts on each node indicate the likelihood of the two SD system types at a given node, and the central node represents the estimated common ancestor. The data and phylogeny underlying the analyses and results displayed in this figure can be found in S1 Data and S1 File, 10.6084/m9.figshare.28562399. (PDF) [file pbio.3003156.s003.pdf]

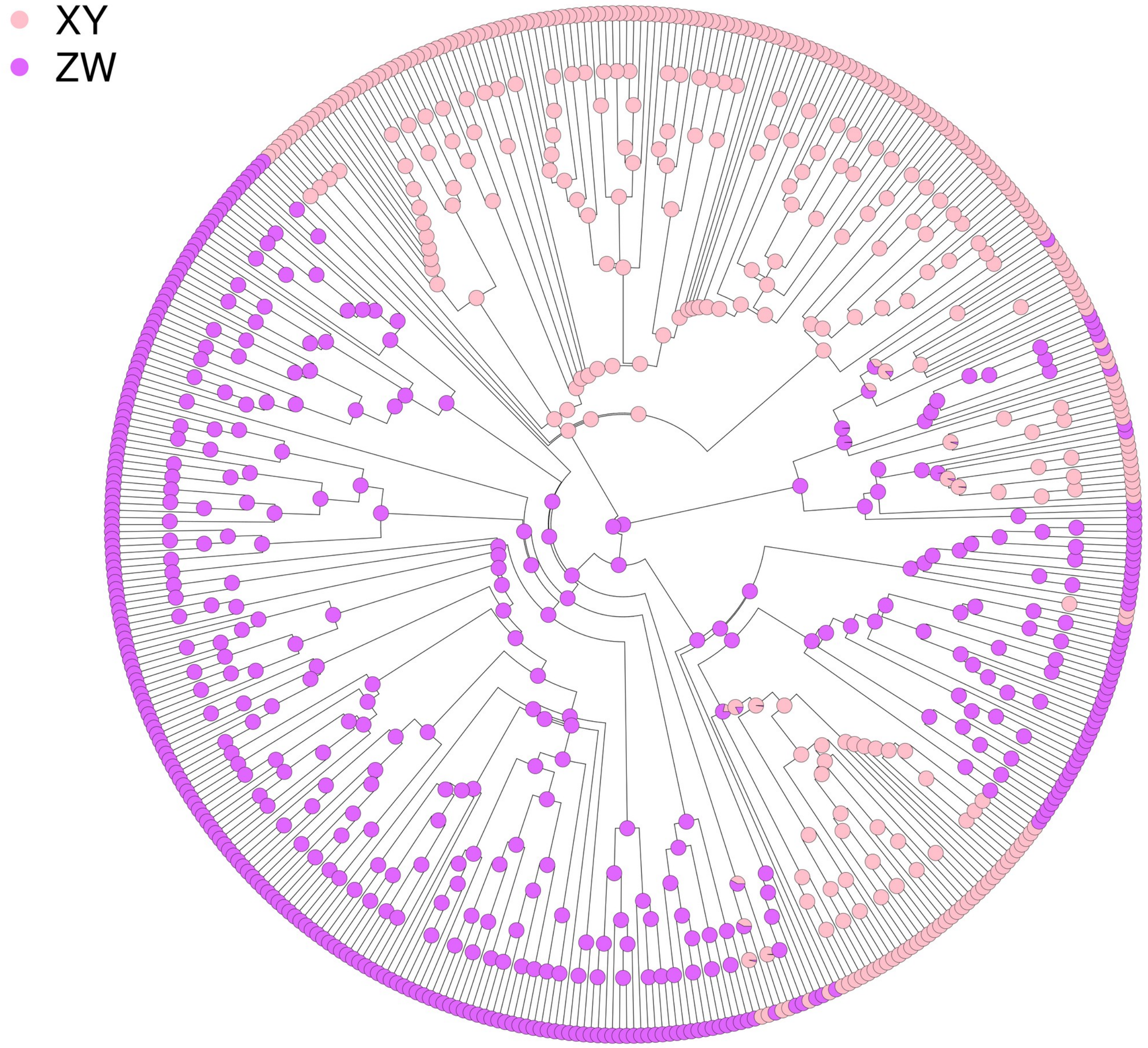

S3 Fig. Result of transition reconstruction between XY and ZW sex determination systems in tetrapod clades included in the study (n=446 species with known sex determination system). We used the ‘make.simmap’ function in the ‘phytools’ package in R with 100 simulations. Result of the "all rates different" (ARD) model type is shown, where different transition rates are allowed between the states, because this model type was supported most by its AIC value. Pie charts on each node indicate the likelihood of the two SD system types at a given node, and the central node represents the estimated common ancestor. The data and phylogeny underlying the analyses and results displayed in this figure can be found in S1 Data and S1 File https://figshare.com/articles/dataset/S1_File_tetrapodtree_Nee_phy/28562399.
