# Supplementary material for: Variation in adult sex ratios in tetrapods is linked to sex chromosomes through mortality differences between males and females
Source: PLoS Biol. 2025 May 12;23(5):e3003156. doi: 10.1371/journal.pbio.3003156 (PMC12148232; doi:10.1371/journal.pbio.3003156)
Supplement: S1 Table — Mean ± SE values estimated from non-standardized data are presented, followed by number of species in brackets. For statistical comparisons among groups, see S3 Table. (PDF) [file pbio.3003156.s004.pdf]

S1 Table. Descriptive statistics for the demographic traits in XY and ZW species in separate taxonomic groups and overall in tetrapods. Mean ± SE values estimated from non-standardised data are presented, followed by number of species in brackets. For statistical comparisons among groups, see S3 Table.

		amphibians*	reptiles	birds	mammals	tetrapods
Adult sex ratio	**XY**	0.512 ± 0.014 (30)	0.478 ± 0.011 (44)		0.417 ± 0.010 (124)	0.443 ± 0.007 (198)
	**ZW**	0.608 ± 0.028 (12)	0.555 ± 0.013 (49)	0.545 ± 0.006 (187)		0.550 ± 0.006 (248)
Birth sex ratio	**XY**	0.387 ± 0.047 (2)	0.485 ± 0.018 (11)		0.514 ± 0.013 (27)	0.500 ± 0.011 (40)
	**ZW**	---	0.512 ± 0.014 (17)	0.509 ± 0.006 (55)		0.510 ± 0.005 (72)
Juvenile mortality bias	**XY**	---	0.011 ± 0.025 (6)		0.023 ± 0.026 (36)	0.021 ± 0.023 (42)
	**ZW**	-0.199 (1)	0.002 ± 0.024 (11)	-0.002 ± 0.019 (52)		-0.004 ± 0.016 (64)
Adult mortality bias	**XY**	0.016 ± 0.024 (10)	0.033 ± 0.038 (23)		0.109 ± 0.023 (52)	0.0758 ± 0.019 (85)
	**ZW**	-0.404 ± 0.115 (5)	0.013 ± 0.020 (18)	-0.044 ± 0.011 (128)		-0.037 ± 0.011 (151)
Maturation bias	**XY**	-0.045 ± 0.020 (20)	-0.029 ± 0.029 (33)		0.037 ± 0.015 (84)	0.009 ± 0.012 (137)
	**ZW**	-0.042 ± 0.024 (8)	-0.108 ± 0.023 (37)	0.018 ± 0.009 (175)		-0.006 ± 0.009 (220)

* Additionally, there are species with unknown type of genetic sex determination among amphibians in our dataset, not included in this table (2, 7, and 9 species for juvenile mortality bias, adult mortality bias, and maturation bias, respectively).
