# Supplementary material for: Variation in adult sex ratios in tetrapods is linked to sex chromosomes through mortality differences between males and females
Source: PLoS Biol. 2025 May 12;23(5):e3003156. doi: 10.1371/journal.pbio.3003156 (PMC12148232; doi:10.1371/journal.pbio.3003156)
Supplement: S2 Table — Results correspond to the models presented in Table 1 of the main text. In (A), where GSD differences in demographic predictors were tested, effect sizes can be interpreted as Cohen’s d, and simulations were run with effect sizes of 0.2, 0.5, and 0.8 representing small, medium, and large effect sizes, respectively. Sample sizes in parentheses show the number of XY and ZW species, respectively. In (B), where demographic predictors of ASR were tested, effect sizes can be interpreted as correlation “b” values, and simulations were run with effect sizes of 0.1, 0.3, and 0.5 reflecting weak, medium, and large correlation effects, respectively. (PDF) [file pbio.3003156.s005.pdf]

S2 Table. Power calculated for bivariate statistical models. Results correspond to the models presented in Table 1 of the main text. In (A), where GSD differences in demographic predictors were tested, effect sizes can be interpreted as Cohen's d, and simulations were run with effect sizes of 0.2, 0.5, and 0.8 representing small, medium, and large effect sizes, respectively. Sample sizes in parentheses show the number of XY and ZW species, respectively. In (B), where demographic predictors of ASR were tested, effect sizes can be interpreted as correlation "b" values, and simulations were run with effect sizes of 0.1, 0.3, and 0.5 reflecting weak, medium, and large correlation effects, respectively.

	**Sample size**	**Estimated effect size ± SE**	**Simulated power, with phylogenetic control**			**Simulated power, without phylogenetic control**		
			**small effect**	**medium effect**	**large effect**	**small effect**	**medium effect**	**large effect**
**(A) Responses to GSD**								
**Adult sex ratio**	446 (198/248)	0.784 ± 0.166	0.278	**0.936**	**1**	**0.782**	**1**	**1**
Birth sex ratio	112 (40/72)	0.072 ± 0.201	0.074	0.188	0.428	0.149	0.595	**0.941**
Juvenile mortality bias	106 (42/64)	-0.202 ± 0.317	0.062	0.16	0.388	0.170	0.683	**0.976**
**Adult mortality bias**	238 (87/151)	-0.508 ± 0.236	0.148	0.572	**0.922**	0.37	**0.965**	**1**
Maturation bias	357 (137/220)	-0.342 ± 0.215	0.194	**0.786**	**0.996**	0.466	**0.996**	**1**
**(B) Predictors of ASR**								
Birth sex ratio	112	0.097 ± 0.093	0.192	**0.804**	**0.998**	0.178	**0.77**	**0.994**
**Juvenile mortality bias**	108	-0.256 ± 0.098	0.162	**0.782**	**0.988**	0.172	**0.742**	**0.992**
**Adult mortality bias**	243	-0.193 ± 0.059	0.368	**0.994**	**1**	0.360	**0.996**	**1**
**Maturation bias**	365	-0.193 ± 0.046	0.534	**1**	**1**	0.486	**1**	**1**

Power calculations with phylogenetic control in both (A) and (B), and without phylogenetic control in (B), were conducted in R using an implementation for linear models (est_lm_power_slope function, published at https://public.wsu.edu/~jesse.brunner/classes/bio572/Lab5_SimulationAndPower.html). Power calculations without phylogenetic control in (A) were conducted using an implementation by Jordan (2021) that allows the settings of sample sizes, sample means and SD separately for the two groups (in our case XY and ZW species) to be compared.
