# Supplementary material for: Variation in adult sex ratios in tetrapods is linked to sex chromosomes through mortality differences between males and females
Source: PLoS Biol. 2025 May 12;23(5):e3003156. doi: 10.1371/journal.pbio.3003156 (PMC12148232; doi:10.1371/journal.pbio.3003156)
Supplement: S3 Table — (A) between each examined demographic variable separately and GSD type across tetrapods, (B) between ASR and each examined demographic variable across tetrapods, Table shows the results of bivariate PGLS models. In section A, GSD type is the predictor, and demographic variables are the response variables, separately each. In section (B), the response variable is the ASR in all models. λ is the phylogenetic signal (Pagel’s lambda), N shows the number of species. Statistically significant (i.e., p < 0.05) relationships are highlighted in bold. (PDF) [file pbio.3003156.s006.pdf]

S4 Table. Associations without the potential influential data points (see Methods for details). A) between ASR and each examined demographic trait across tetrapods, B) between ASR and the interaction of demographic traits and GSD type. Table shows the results of bivariate PGLS models (A), and results of PGLS models including the two-way interaction of each demographic trait with GSD type (B). **λ** is the phylogenetic signal (Pagel's lambda), N shows the number of species, N_{excl} shows the number of excluded influential data points for each model. Note that the continuous variables were standardised to 0 mean and 1 SD, so the model coefficients (b) are comparable across predictors. Statistically significant (i.e. $p<0.05$) relationships are highlighted in bold.

**Predictor variable**	**b ± SE**	**t**	**p**	**λ**	**N**	**N_{excl}**
**A)**						
**Birth sex ratio**		No influential points detected				
**Juvenile mortality bias**	-0.304 ± 0.122	-2.491	**0.014**	0.324	106	2
**Adult mortality bias**	-0.351 ± 0.069	-5.064	**< 0.001**	0.263	238	4
**Maturation bias**	-0.183 ± 0.057	-3.195	**0.002**	0.383	356	8
**B)**	**F**	**df**	**p**	**λ**	**N**	**N_{excl}**
**Birth sex ratio × GSD**	3.385	1;106	*0.069*	0.359	110	2
**Juvenile mortality bias × GSD**	5.526	1;100	**0.021**	0.063	104	2
**Adult mortality bias × GSD**	0.807	1;227	0.369	0.172	231	7
**Maturation bias × GSD**	1.332	1;347	0.249	0.308	351	6

In A) "b" shows the slope of the relationship between ASR and demographic variables.
In B) and C), results of type 3 ANOVA are presented.

Excluded species for bivariate models: Birth sex ratio: no influential points detected; Juvenile mortality bias: Loxioides_bailleui, Bison_bison; Adult mortality bias: Tayassu_pecari, Crocuta_crocuta, Ctenosaura_melanosterna, Pogonocichla_stellata; Maturation bias: Antidorcas marsupialis, Hynobius leechii, Cisticola_juncidis, Tetrao_urogallus, Ameiva_quadrilineata, Anolis_garmani, Apalone_spinifera, Vipera_aspis

Excluded species for GSD interactions: Birth sex ratio: Megalurus_gramineus, Iberolacerta_aranica; Juvenile mortality bias: Loxioides_bailleui, Bison_bison; Adult mortality bias: Tayassu_pecari, Crocuta_crocuta, Ctenosaura_melanosterna, Pogonocichla_stellata, Circus_cyaneus, Selasphorus_platycercus, Bufo_bufo; Maturation bias: Cisticola_juncidis, Tetrao_urogallus, Ameiva_quadrilineata, Anolis_garmani, Apalone_spinifera, Vipera_aspis.

Excluded species for Taxon interactions: Birth sex ratio: Megalurus_gramineus (bird); Juvenile mortality bias: Loxioides_bailleui (bird), Bison_bison (mammal); Adult mortality bias: Tayassu_pecari (mammal), Crocuta_crocuta (mammal), Ctenosaura_melanosterna (reptile), Pogonocichla_stellata (bird), Circus_cyaneus (bird), Selasphorus_platycercus (bird); Maturation bias: Hynobius leechii (amphibian), Cisticola_juncidis (bird), Tetrao_urogallus (bid), Jacana spinosa (bird), Ameiva_quadrilineata (reptile).

Note that amphibians are excluded form models of birth sex ratio and juvenile mortality bias due to lack of enough data for this group.
