# Supplementary material for: Variation in adult sex ratios in tetrapods is linked to sex chromosomes through mortality differences between males and females
Source: PLoS Biol. 2025 May 12;23(5):e3003156. doi: 10.1371/journal.pbio.3003156 (PMC12148232; doi:10.1371/journal.pbio.3003156)
Supplement: S5 Table — Continuous variables are standardized in all models. Slopes of the relationships for each group were taken from models that include interaction between demographic traits and GSD type (XY or ZW). Estimates are based on models in which continuous variables were standardized to 0 mean and 1 SD. Slopes significantly differing from zero, i.e., with 95% confidence interval (CI) excluding zero, are highlighted in bold. λ is the phylogenetic signal (Pagel’s lambda), and N shows the number of species in each model, and the number of species in each presented group (in italics). (PDF) [file pbio.3003156.s008.pdf]

S5 Table. Relationships between ASR and demographic traits separately for XY and ZW species. Continuous variables are standardised in all models. Slopes of the relationships for each group were taken from models that include interaction between demographic traits and GSD type (XY or ZW). Estimates are based on models in which continuous variables were standardised to 0 mean and 1 SD. Slopes significantly differing from zero, i.e. with 95% confidence interval (CI) excluding zero, are highlighted in bold. **λ** is the phylogenetic signal (Pagel's lambda), and N shows the number of species in each model, and the number of species in each presented group (in italics).

Predictor	Slope ± SE	95% CI	λ	N
Comparing the ASR - demography relationships between XY and ZW species				
**Birth sex ratio**			0.352	112
GSD type: XY	0.121 ± 0.125	-0.127 ; 0.370		*40*
GSD type: ZW	0.017 ± 0.140	-0.261 ; 0.296		*72*
**Juvenile mortality bias**			0.057	106
GSD type: XY	-0.059 ± 0.133	-0.324 ; 0.260		*42*
GSD type: ZW	**-0.435 ± 0.123**	**-0.679 ; -0.191**		*64*
**Adult mortality bias**			0.184	238
GSD type: XY	-0.107 ± 0.080	-0.265 ; 0.051		*87*
GSD type: ZW	**-0.353 ± 0.083**	**-0.516 ; -0.190**		*151*
**Maturation bias**			0.307	357
GSD type: XY	-0.091 ± 0.069	-0.227 ; 0.045		*137*
GSD type: ZW	**-0.224 ± 0.062**	**-0.345 ; -0.102**		*220*
