# Supplementary material for: Variation in adult sex ratios in tetrapods is linked to sex chromosomes through mortality differences between males and females
Source: PLoS Biol. 2025 May 12;23(5):e3003156. doi: 10.1371/journal.pbio.3003156 (PMC12148232; doi:10.1371/journal.pbio.3003156)
Supplement: S6 Table — SE is only estimated for non-standardized coefficients in ‘piecewiseSEM’. (PDF) [file pbio.3003156.s009.pdf]

**S6 Table.** Estimates of non-standardised and standardised coefficients, and associated SE and p-values for paths in the most supported path model (Model 1.b) in the analysis by 'piecewiseSEM'. SE are only estimated for non-standardised coefficients in 'piecewiseSEM'.

| **Path** | **Non-standardised path coefficient ± *SE*** | **Standardised path coefficient** | ***Z*** | ***p*** |
|---|---|---|---|---|
| GSD → ASR | 0.105 ± 0.054 | 0.182 | 1.937 | *0.056* |
| GSD → AMB | -0.153 ± 0.080 | -0.197 | - 1.922 | *0.058* |
| JMB → ASR | - 0.207 ± 0.081 | -0.242 | - 2.551 | **0.013** |
| AMB → ASR | - 0.158 ± 0.071 | -0.214 | - 2.217 | **0.029** |
| MAT → ASR | - 0.242 ± 0.094 | -0.235 | - 2.559 | **0.012** |
