# Supplementary material for: Variation in adult sex ratios in tetrapods is linked to sex chromosomes through mortality differences between males and females
Source: PLoS Biol. 2025 May 12;23(5):e3003156. doi: 10.1371/journal.pbio.3003156 (PMC12148232; doi:10.1371/journal.pbio.3003156)
Supplement: S7 Table — ∆AIC values show the difference between the model including a quadratic term of each demographic predictor and the model without any quadratic term (i.e., positive ∆AIC value means lower AIC value for the model without the quadratic term). P-values show the result of a likelihood ratio test between the two corresponding models with and without the quadratic term, where significant (<0.05) p-value suggests better fit for models with lower AIC (i.e., without quadratic terms). (PDF) [file pbio.3003156.s010.pdf]

S7 Table. Testing the quadratic effect of demographic predictors on ASR. ΔAIC values show the difference between the model including a quadratic term of each demographic predictor and the model without any quadratic term (i.e. positive ΔAIC value means lower AIC value for the model without the quadratic term). P-values show the result of a likelihood ratio test between the two corresponding models with and without the quadratic term, where significant (<0.05) p-value suggests better fit for models with lower AIC (i.e., without quadratic terms).

Predictor	**ΔAIC**	**p**
**Birth sex ratio**	6.297	0.038
**Juvenile mortality bias**	6.288	0.038
**Adult mortality bias**	7.479	0.019
**Maturation bias**	8.342	0.012
